# Editorial: Infants' Understanding and Production of Goal-Directed Actions in the Context of Social and Object-Related Interactions

**DOI:** 10.3389/fpsyg.2017.00787

**Published:** 2017-05-16

**Authors:** Daniela Corbetta, Jacqueline Fagard

**Affiliations:** ^1^Director Infant Perception-Action Laboratory, Department of Psychology, University of TennesseeKnoxville, TN, USA; ^2^Centre National de la Recherche Scientifique, University Paris DescartesParis, France

**Keywords:** infants reaching, goal-directed actions, social cognition, action planning, action understanding

Goal-directed actions are central to our everyday lives. They are also considered a hallmark of prospective control. Indeed, in order to attain an intended goal (whether a wanted object or an aimed location in space), a certain amount of anticipation and forward planning is necessary. In humans, early signs of anticipation of actions can be detected as early as *in utero* (Myowa-Yamakoshi and Takeshita, 2006; Zoia et al., 2007), but significant changes in goal-directed actions happen in infancy, specifically around the age of 3–5 months, when infants start to produce their first reaching attempts toward an object in their vicinity (Thelen et al., 1993; Corbetta et al., 2014). Other precursor behaviors of anticipation and goal-directedness can also be seen in the previous months or weeks preceding reach onset, and occur in the form of anticipatory gaze responses or tracking of visual events (see for example, Haith et al., 1988; Wentworth and Haith, 1992; Hofsten and Rosander, 1997; Agyei et al.). The action of reaching, however, is the first behavior in early infancy that brings together a deliberate movement of the hand toward an object-goal visually attended.

The emergence of reaching undeniably marks an important milestone in early development. This has been acknowledged for decades. But in recent years, it has been discovered that the emergence of reaching triggers a rapid developmental cascade that has far and wide implications for infants' cognitive and sensorimotor development. From about 12 weeks of age, infants already begin to display predictive looking behaviors and pick up cues from moving objects (Rosander and von Hofsten, 2004; Agyei et al.). As reaching emerges, they learn to pick up even more sophisticated cues to direct their actions within their surroundings, scenes, and people in their immediate social environment (Fagard et al.; Fantasia et al.; Filippi and Woodward; Williams and Corbetta), they learn to plan their movements accordingly (Fantasia et al.; Williams and Corbetta), and they also begin to understand other people's actions and intentions in the context of social interactions (Zmyj et al.; Filippi and Woodward; Robson and Kuhlmeier).

The goal of this research topic is to bring together these exciting and recent developments. Much of the manuscripts in this research topic address novel aspects of infants' production and understanding of goal-directed actions in relation to object and others. The first half of the articles focus on the origins, changes, and brain activities related to the prospective control of infants' own goal-directed actions. Two articles discuss the very first behaviors that are preceding and subsequently leading to the formation goal-directed actions. Agyei et al. review the early development of prospective control in infancy, particularly in the case of visual motion perception, while Thomas et al. focus on the spontaneous self-touching behaviors in the first 6 months of life that form the foundation of goal-directed reaching and grasping. Williams and Corbetta center on the emergence of reaching and investigate how novice reachers quickly learn from the consequences of their own actions to modulate their arm response depending on whether they are aiming for a continuously moving target or a target that moves only in response to successful contacts with the object target. Both Agyei et al. and Kaur et al. extend these research questions to special populations. Agyei et al. report preliminary differences in prospective control between full-term and pre-term infants, and Kaur et al. show that over the 2 first years of life, infants at risk of Autism Spectrum Disorder show less manual exploratory behaviors of objects compared to same age typically developing infants. Two papers also use brain imaging techniques to capture developmental changes in goal-directed behaviors. Agyei et al. illustrate how Visual Evoked Potentials (captured via EEG) can reveal impaired functioning in the dorsal visual stream of preterm infants, while Nishiyori demonstrates how functional near-infrared spectroscopy (fNIRS) can be a useful tool to capture cortical activity as infants develop goal-directed actions (see also Nishiyori et al., 2016).

The second half of the papers in this research topic focus more specifically on how infants understand intents and goal-directed actions performed by others. Since the discovery of the mirror neuron (Buccino et al., 2001) there has been growing evidence showing that infants' emerging ability to reach influences not only their ability to act on their environment, but also their ability to understand and anticipate the actions of others (e.g., Falck-Ytter et al., 2006; Cannon et al., 2012). Several contributions in this research topic build on these prior findings. Fantasia et al., for instance, provide compelling evidence that at 3-months-old infants already, not only plan ahead and adjust their posture in preparation for being picked up by their caregiver (Reddy et al., 2013), but show also sensitivity to change in timing in the pick-up sequence as provided by the adult. To older infants, Fagard et al. presented a variety of tool use demonstrations (modeled by an adult) to retrieve an object out of reach. They find that when the actor first displays its intentions, infants are more likely to learn how to use the tool successfully than when a simple demonstration of how to use the tool is provided. Clearly, infants use intent-based cues provided by their social partner to position themselves in the interaction or solve problems. The way infants use social cues provided by a partner in the context of object-directed actions to attribute goals and understand others action is elegantly reviewed by Robson and Kuhlmeier. We know also that the cue details that infants pick up from social actors can be quite specific. Filippi and Woodward show that infants with anticipatory reaching experience can also visually anticipate the goal-object being reached by a partner based on the orientation of their hand and its congruency with the object goal orientation. Finally, a study from Zmyj et al. examines the extent to which older infants understanding of false-beliefs (perpetrated by an agent) is related to their working memory. They test infants in two tasks designed to assess working memory but find no relations between tasks and thus no direct relation between working memory and false-belief understanding.

In conclusion, the ensemble of research showcased in this research topic capture the breath of the questions that relate to infants' understanding and production goal-directed actions in the context of social and object-related interactions.

## Author contributions

All authors listed, have made substantial, direct and intellectual contribution to the work, and approved it for publication.

## Funding

This work was supported by NSF1229176 and NICHD R21HD065042 to DC and ANR-13-BSH2-0007-01 to JF.

## Conflict of interest statement

The authors declare that the research was conducted in the absence of any commercial or financial relationships that could be construed as a potential conflict of interest.
